# The Impact of Early Oral Feeding on Post‐Operative Morbidity After Esophagectomy: A Systematic Review and Meta‐Analysis

**DOI:** 10.1002/wjs.70417

**Published:** 2026-05-24

**Authors:** Debasri Jena, Lixuan Feng, Kwaku Addo‐Osafo, Armin Rouhi, Janice Y. Kung, Sukhdeep Jatana, Kevin Verhoeff, Uzair Jogiat, Simon R. Turner, Eric L. R. Bédard

**Affiliations:** ^1^ Division of Thoracic Surgery University of Alberta Edmonton Alberta Canada; ^2^ Department of General Surgery University of Alberta Edmonton Alberta Canada; ^3^ Geoffrey and Robyn Sperber Health Sciences Library University of Alberta Edmonton Alberta Canada

**Keywords:** anastomotic leak, early oral feeding, esophagectomy, postoperative morbidity

## Abstract

**Background:**

Anastomotic leak and post‐operative pneumonia are major contributors to postoperative morbidity following esophagectomy and have traditionally led to delays in oral feed initiation. Newer evidence, however, suggests early initiation of feeds may be safe. The aim of this systematic review and meta‐analysis is to assess the safety of initiation of oral feeds on postoperative day one versus delayed initiation.

**Methods:**

A systematic search was conducted on six databases and studies were included if they compared safety outcomes of adult esophagectomy patients initiating feeds on postoperative day one versus delayed imitation. A random‐effects meta‐analysis with restricted maximum likelihood was performed and study quality was assessed with the Newcastle‐Ottawa Scale.

**Results:**

Ten studies with a total of 1714 patients were included. Our primary outcome of anastomotic leak showed no significant between the early and delayed oral feeding group (8 studies, OR 0.92, 95% CI 0.59–1.41, *p* = 0.69). For the secondary outcomes, there was no difference in length of stay (4 studies, mean difference −2.27 days, 95% CI −5.13 to 0.60, *p* = 0.12) and there was decreased odds of postoperative pneumonia (9 studies, OR 0.74, 95% CI 0.58–0.95, *p* = 0.02).

**Conclusions:**

Initiating oral intake on postoperative day one after esophagectomy does not increase length of stay or risk of anastomotic leak and postoperative pneumonia. These findings support consideration of early oral feeding as a safe component of perioperative care when applied appropriately.

## Graphical abstract

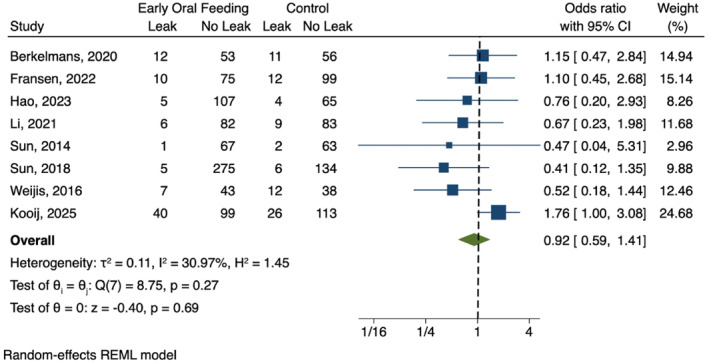

## 1 Introduction

The incidence of malnutrition amongst esophageal cancer patients is estimated between 60% and 85% [1, 2]. Malnutrition significantly reduces the tolerance of treatment while increasing the risk of surgical complications [3, 4]. Treatment is determined based on a variety of factors including histological subtype, clinical staging, tumor location, and patient factors, but often includes surgical resection accompanied by neoadjuvant or adjuvant therapies [5, 6, 7]. Although improvements have been made, esophagectomies have one of the highest mortality and morbidity rates among all surgical procedures with complication rates up to 60% [1, 8]. Despite numerous approaches to reduce anastomotic complications, anastomotic leak persists as a feared complication occurring in approximately 10%–20% of cases [2, 3, 9, 10, 11, 12, 13].

Enhanced recovery after surgery (ERAS) programs aim to improve outcomes associated with surgeries and guidelines specific to esophagectomy have been developed [14, 15]. The guidelines provide an evidence‐based approach to management of patients undergoing esophagectomy, but adherence and implementation of recommendations is still heterogenous. Specifically, the removal of nasogastric tubes on post‐operative day two, is only a part of practice among 63.9% of responders and early oral feeding with a fluid diet only practiced among 50.8% [15]. This hesitancy to adopt protocols for esophagectomy could be due to the substantial fear of leaks, as historical standard of care for nutritional support after esophagectomy included a strict nil per os period of five to 7 days to allow for healing of the anastomosis between the upper esophagus and conduit [16, 17].

In the literature, many studies have evaluated whether early feeding increases complications after esophagectomy [4, 5, 6, 7]. The definition of early oral feeding is a shifting paradigm as what was considered early 20‐year ago is now standard practice. Thus, it is unclear whether early oral feeding may be associated with complications after esophagectomy. Given the paradigm shift toward enhanced recovery after surgery (ERAS) protocols and the recently published prospective studies in the literature, an up‐to‐date systematic review and meta‐analysis with a focus on early oral feeding defined as post‐operative day one is warranted.

The purpose of this systemic review and meta‐analysis was to provide an updated review on early oral feeding after esophagectomy focusing specifically post‐operative morbidity and length of stay following oral intake starting post‐operative day one. A better understanding of these outcomes may provide evidence to inform current surgical practice and lead to better patient outcomes.

## 2 Methods

### 2.1 Statement of Human Rights

This systematic review and meta‐analysis did not require research ethics board review.

### 2.2 Sex/Gender Reporting

When reported, sex distribution was extracted. However, studies were not excluded based on sex.

**Use of Artificial Intelligence**: Artificial Intelligence and Large Language Models were not used in this study.

### 2.3 Search Strategy

The reporting of this systematic review was guided by the standards of the Preferred Reporting Items for Systematic Review and Meta‐Analysis (PRISMA) Statement (Supporting Information S2: Online Resource 1). A medical librarian (JYK) developed and executed comprehensive searches in Ovid MEDLINE (1946–2025), Ovid Embase (1974–2025), Scopus, Web of Science Core Collection, and Cochrane Library (via Wiley) on May 30th, 2023, and updated on October 2^nd^, 2025. No language or date limits were applied. Refer to Supporting Information S1: Online Resource 2 for the full search strategies. In addition to subscription databases, the research team reviewed the first 200 results in Google Scholar [18]. Bibliographies from included studies were also reviewed.

### 2.4 Study Outcomes

The primary objective of this study was to determine if early oral feeding is associated with a higher incidence of anastomotic leak after esophagectomy. A secondary objective of this study was to evaluate the incidence of post‐operative pneumonia and length of stay.

### 2.5 Study Selection and Data Extraction

Studies which involved patients undergoing esophagectomy, applied early oral feeding strategies, and measured post‐operative morbidity and length of stay were included. Early oral feeding was defined as the initiation of oral intake on post‐operative day one. Additional inclusion criteria were: age greater than 18 years and studies with greater than five patients. Animal studies, case reports, case series, poster presentations, and conference proceedings with insufficient detail were excluded. Additionally, studies which did not include a comparator group were also excluded. Two primary researchers evaluated titles and abstracts (DJ and LF), with potentially eligible studies undergoing a full‐text review by two primary researchers (DJ and LF). Upon updating of the search, two primary researchers evaluated titles and abstracts (UJ and AR), with potentially eligible studies undergoing a full‐text review by two primary researchers (UJ and AR). Disagreements in the study inclusion or exclusion criteria between the two primary researchers were resolved by discussion and consensus among the authorship group.

For studies that met the inclusion criteria, data was extracted regarding study characteristics including author, year of publication, study design, baseline patient demographics, early oral feeding strategy, type of esophagectomy performed, indication for esophagectomy, and characterization of malignancy (if available).

### 2.6 Risk of Bias Assessment

The methodological quality of the studies was assessed by two independent reviewers (DJ and LF) using the Newcastle‐Ottawa Scale (NOS).

Publication bias was evaluated using a funnel plot generated in Stata 17.0, which was visually inspected for symmetry. Asymmetry was suspected if the individual study estimates were not evenly distributed around the weighted overall effect estimate.

### 2.7 Statistical Analysis

Descriptive categorical data were expressed as percentages and continuous data were expressed as weighted means, where appropriate. Meta‐analysis was performed to evaluate the difference in the incidence of anastomotic leak, post‐operative pneumonia, and length of stay among patients receiving early oral feeding compared to standard of care. Estimated effects were calculated using Stata 17.0 with a restricted maximum likelihood random‐effects model with effect measures reported as odds ratios for anastomotic leak and post‐operative pneumonia and mean difference for length of stay. Heterogeneity was calculated using the L'Abbé plot and the *I* ^2^ statistics. Low heterogeneity was classified as less than 25%, moderate as 25% and 75%, and high as greater than 75%. Tests for statistical significance were two‐tailed with significant *p*‐values defined as < 0.05 a priori.

## 3 Results

### 3.1 Study Selection

A total of 1179 articles were identified after removal of duplicates and underwent abstract screening. Of these, 1105 records were excluded after title and abstract screening for not meeting inclusion criteria, leaving 74 articles for full‐text review. Ten studies met the final inclusion criteria [6, 14, 16, 19, 20, 21, 22, 23, 24, 25]. The search and study inclusion flow diagram is available in Supporting Information S3: Online Resource 3. Due to the variability in definition of early oral feeding among the manuscripts, the meta‐analysis was restricted to initiation of early oral feeding on post‐operative day zero or one. This resulted in eight studies included in the meta‐analysis for anastomotic leak [6, 14, 19, 20, 22, 23, 24, 25], nine studies include for post‐operative pneumonia [6, 14, 19, 20, 21, 22, 23, 24, 25], and four studies included in the meta‐analysis for length of stay [16, 19, 23, 25]. Table 1 provides the patient and study characteristics of the individual studies. Table 2 provides the feeding strategies adopted for early and delayed oral feeding in each study. After completion of the initial review, one additional study published in 2025 by Kooij et al. met the inclusion criteria [25]. As a consequence of this, the systematic search was re‐run, as detailed in Section 2.1 and there were no additional studies identified. The additional study was incorporated into the updated analyses, resulting in a total of 10 studies included in this review.

**TABLE 1:** Baseline characteristics of included studies.

Study, year	Study design	Sample size (*n*, %)	Age, years	Histology	Type of esophagectomy	Inclusion/Exclusion criteria
Sun 2015 [19]	PCS	EOF: 68 (51.1%) DOF: 65 (48.9%)	61.1 ± 8.4 (EOF) 60.3 ± 7.0 (DOF)	SCC	MIE	Inclusion: Histologically confirmed SCC, age < 80, MIE planned, adequate organ function, no prior chemo/radiotherapy. Exclusion: Severe comorbidities or malnutrition (BMI < 15).
Weijs 2016 [20]	PCS	EOF: 50 (50.0%) DOF: 50 (50.0%)	67 [IQR 60–71] (EOF) 65 [IQR 58–71] (DOF)	SCC/AC grouped	MIE	Inclusion: Age ≥ 18 years; undergoing IL‐MIE. Exclusion: Preoperative weight loss > 15%, intellectual disability, or inability to take oral intake.
Berkelmans 2018 [21]	PCS	EOF: 57 (43.5%) DOF: 74 (56.5%)	67 [IQR 60–71] (EOF) 66 [IQR 58–71] (DOF)	SCC/AC grouped	ILE–MIE	Inclusion: Age ≥ 18 years; underwent IL‐MIE; ≥ 3 weight measurements available. Exclusion: ≥ 3 missing weight measurements.
Sun 2018 [6]	PCS	EOF: 140 (50.0%) DOF: 140 (50.0%)	63 [IQR 58–68] (EOF) 63 [IQR 58–69] (DOF)	SCC/AC grouped (majority SCC)	McKeown–MIE	Inclusion: Consecutive patients ≥ 18 years undergoing McKeown MIE. Exclusion: Age ≥ mk80, tumor precluding MIE, severe comorbidity, hepatocirrhosis, diabetes with organ damage, exploratory surgery, ICU > 24h, bilateral RLN injury.
Berkelmans 2020 [22]	RCT	EOF: 65 (49.2%) DOF: 67 (50.8%)	65 [IQR 59–70] (EOF) 65 [IQR 61–70] (DOF)	SCC	ILE–MIE	Inclusion: Age ≥ 18; planned IL‐MIE. Exclusion: Unable to tolerate oral intake, KPS < 80, malnutrition (> 15% weight loss), metastases, total gastrectomy.
Li 2021 [23]	RCS	EOF: 821 (51.5%) DOF: 774 (48.5%)	32–85 (EOF) 38–85 (DOF)	SCC/AC grouped	ILE, McKeown–MIE	Inclusion: Esophagectomy without distant metastasis or severe dysfunction. Exclusion: Palliative surgery or other malignancies.
Fransen 2022 [14]	PCS	EOF: 85 (43.4%) DOF: 111 (56.6%)	65 [IQR 58–70] (EOF) 67 [IQR 61–74] (DOF)	NR	MIE	Inclusion: Age ≥ 18, IL‐MIE. Exclusion: KPS < 80, > 15% weight loss, transhiatal/McKeown, salvage, metastatic disease, conflicting trial participation.
Yang 2022 [16]	RCS	EOF: 200 (63.5%) DOF: 115 (36.5%)	62.9 ± 8.1 (EOF) 62.6 ± 7.9 (DOF)	NR	McKeown–MIE	Inclusion: Pathology‐confirmed carcinoma, age 18–75. Exclusion: Exploratory/emergency/robotic surgery, severe organ disease, major postoperative complications, missing data.
Hao 2023 [24]	RCS	EOF: 112 (61.9%) DOF: 69 (38.1%)	63 [IQR 56–68] (EOF) 61 [IQR 56–68] (DOF)	SCC/AC grouped	McKeown	Inclusion: Primary esophageal cancer, first‐time treatment, neoadjuvant chemo, McKeown MIE. Exclusion: Laparotomy, incomplete MIE, ICU > 24h, RLN injury, other therapy.
Kooij 2025 [25]	RCS	EOF: 139 (50.0%) DOF: 139 (50.0%)	66 ± 9 (EOF) 66 ± 9 (DOF)	SCC/AC grouped	MIE	Inclusion: Adults undergoing esophagectomy with gastric conduit reconstruction. Exclusion: Non‐cancer, missing data, non‐hand‐sewn anastomosis.

Abbreviations: AC, adenocarcinoma; DOF, delayed oral feeding; EOF, early oral feeding; IL, Ivor‐Lewis; MIE, minimally invasive esophagectomy; PCS, prospective cohort study; RCS, retrospective cohort study; RCT, randomized controlled trial; SCC, squamous cell carcinoma.

**TABLE 2:** Feeding strategies for early oral feeding (EOF) and delayed oral feeding (DOF).

Study, year	EOF protocol (stepwise)	DOF protocol (stepwise)
Sun 2015 [19]	Liquid diet started on POD1 if no delayed gastric emptying; full liquids by POD8.	Nil‐by‐mouth with enteral feeding for first 7 days; increase rate; if no leak, full liquids from POD8.
Weijs 2016 [20]	POD0 clear liquids; POD1–6 liquid diet; solids from POD7; enteral if oral insufficient.	Nil‐by‐mouth with enteral nutrition for 4–7 days; oral intake delayed until POD4–7.
Berkelmans 2018 [21]	POD1 oral liquids increasing to solids by POD5; start enteral only if < 50% caloric needs met.	Enteral feeding POD1; oral intake allowed POD5–14; tube feeds continue until oral intake meets needs.
Sun 2018 [6]	POD1 oral liquids; no routine tube; PN POD1–3 then stop if oral tolerated.	Nasoenteral tube POD1; nil‐by‐mouth until POD7; stop enteral once soft diet tolerated.
Berkelmans 2020 [22]	Clear liquids‐initiated POD 0, progression to solids by POD 15	Clear liquids POD 5, progression to solids by POD19.
Li 2021 [23]	Initiated clear fluids between POD 0–2, transition to semi‐fluids by POD 4.	NPO 2–5 days with NG, then NJ fed and transitioned to PO clear liquids.
Fransen 2022 [14]	Day of surgery: ≤ 250cc sips; POD1 ≥ 500cc liquids; POD5 ≥ s1500cc; solids from POD15.	Day of surgery: ≤ 250cc sips; solids from POD20.
Yang 2022 [16]	POD1 clear fluid stepwise oral intake; PN maintained until oral intake adequate.	POD2–3 stepwise oral gastric decompression < 200 mL/d; PN until intake adequate.
Hao 2023 [24]	POD1 non‐tube solid food with supervised chewing protocol.	POD7 start oral; NG/feeding‐tube and PN until adequate oral intake.
Kooij 2025 [25]	POD0 popsicle; POD1–3 clear liquids; POD4 full liquids.	POD1 jejunostomy enteral feeds; POD1–3 nil‐by‐mouth; POD4 sips; liquid diet thereafter.

Abbreviation: POD, postoperative day.

### 3.2 Study and Patient Characteristics

Out of the 10 included studies, one was a randomized controlled trial, five were prospective cohort studies, and four were retrospective cohort studies. Study years ranged from 2004 to 2025. There was a total of 1714 patients included, 902 (52.6%) were in the early oral feeding cohort and 812 (47.4%) received delayed feeding. The mean age was 63.9 years +/− 8.8 years in the early oral feeding group and 63.7 +/− 8.8 years in the delayed feeding group. Males accounted for approximately 71% of patients, and females accounted for approximately 29% of patients. Early oral intake was defined as initiation of a liquid oral diet on post‐operative day zero or one in all studies (Table 2). This was compared to standard of care at the respective studies' institution, with variability noted in the initiation of oral intake ranging from post‐operative day two to post‐operative day 20, with a mean of approximately postoperative day eight across all studies. Among the included studies, approximately 90% of patients underwent minimally invasive esophagectomy (MIE). Histology was reported in five studies, with 204 (33.8%) patients with adenocarcinoma and 400 (66.2%) with squamous cell carcinoma [6, 17, 20, 21, 24].

### 3.3 Critical Appraisal of Studies

Ten studies were assessed for methodological quality per the Newcastle‐Ottawa Scale (NOS) performed by two reviewers (DJ, LF). All studies demonstrated moderate to high methodological quality, with most studies scoring between eight and nine, suggesting a low risk of bias across domains. Reviewer agreement was high with minimal discrepancies, indicating strong inter‐rater reliability. Table 3 provides the NOS scores for the included studies.

**TABLE 3:** Ottawa new‐castle risk of bias assessment by two reviewers for the included studies.

Study	Reviewer 1 score	Reviewer 2 score	Final score	Conflicts	Selection (0–4)	Comparability (0–2)	Outcome exposure (0–3)
Hao, 2023 [24]	8	8	8	No	4	1	3
Fransen, 2022 [14]	9	9	9	No	4	2	3
Berkelmans, 2020 [22]	9	9	9	No	4	2	3
Berkelmans, 2018 [21]	8	8	8	No	4	1	3
Sun, 2015 [19]	9	9	9	No	4	2	3
Weijs, 2016 [20]	8	8	8	No	4	1	3
Li, 2021 [23]	9	9	9	No	4	2	3
Sun, 2018 [6]	9	9	9	No	4	2	3
Yang, 2022 [16]	8	8	8	No	4	1	3

### 3.4 Anastomotic Leak

**FIGURE 1:**
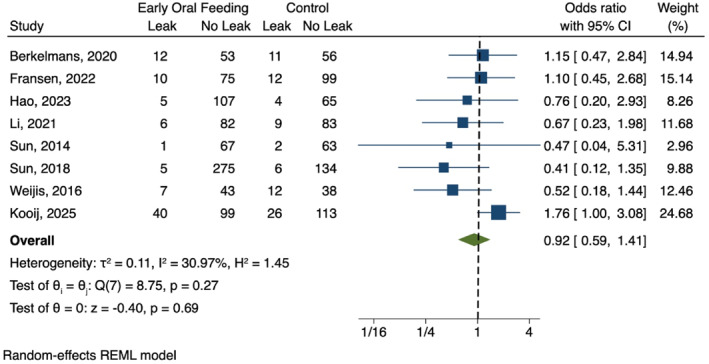
Forrest plot of the random‐effects meta‐analysis for the odds of anastomotic leak among studies comparing early oral feeding group to the control group (delayed oral feeding or standard of care) among patients undergoing esophagectomy.

Eight studies reported incidence of anastomotic leak (Table 4). Pooled meta‐analysis revealed no significant difference in the odds of anastomotic leak among patients in the early oral feeding group compared to standard of care (OR 0.92, 95% CI 0.59 to 1.41, *p* = 0.69) (Figure 1). Heterogeneity was interpreted as moderate based on the *I* ^2^ value of 30.97% and the Galbraith plot demonstrating no studies reporting mean estimates outside of the 95% confidence interval of the regression line (Supporting Information S4: Online Resource 4 and Supporting Information S5: Online Resource 5).

**TABLE 4:** Outcomes after early oral feeding following esophagectomy.

Study, year	Anastomotic leak EOF	Anastomotic leak control	Pneumonia EOF	Pneumonia control	Length of stay EOF	Length of stay control
Sun 2015 [19]	1/68 (1.5%)	2/65 (3.1%)	5/68 (7.4%)	6/65 (9.2%)	9.2 ± 2.6	10.7 ± 3.9
Weijs 2016 [20]	7/50 (14.0%)	12/50 (24.0%)	14/50 (28.0%)	20/50 (40.0%)	12 (M)	13 (M)
Berkelmans 2018 [21]	NR	NR	18/65 (27.7%)	28/67 (41.8%)	9 (M)	9 (M)
Sun 2018 [6]	5/140 (3.6%)	6/140 (4.3%)	15/140 (10.7%)	17/140 (12.1%)	7 [M (7–8 R)]	10 [M (9–12 R)]
Berkelmans 2020 [22]	12/65 (18.5%)	11/67 (16.4%)	16/65 (24.6%)	23/67 (34.3%)	9 (M)	9 (M)
Li 2021 [23]	6/87 (6.9%)	9/92 (9.8%)	14/87 (16.1%)	19/92 (20.7%)	16.3 ± 5.1	22.5 ± 5.0
Fransen 2022 [14]	10/85 (11.8%)	12/111 (10.8%)	24/85 (28.2%)	39/111 (35.1%)	8 [M (7–10)]	10 [M (7–19)]
Yang 2022 [16]	NR	NR	NR	NR	7.8 ± 3.5	10.1 ± 4.0
Hao 2023 [24]	5/112 (4.5%)	4/69 (5.8%)	12/112 (10.7%)	10/69 (14.5%)	8 [M (7–10)]	11 [M (9–15)]
Kooij 2025 [25]	40/139 (28.8%)	26/139 (18.7%)	47/139 (33.8%)	46/139 (33.1%)	12 ± 8.9	11 ± 5.4

Abbreviation: NR, not reported.

### 3.5 Post‐Operative Pneumonia

**FIGURE 2:**
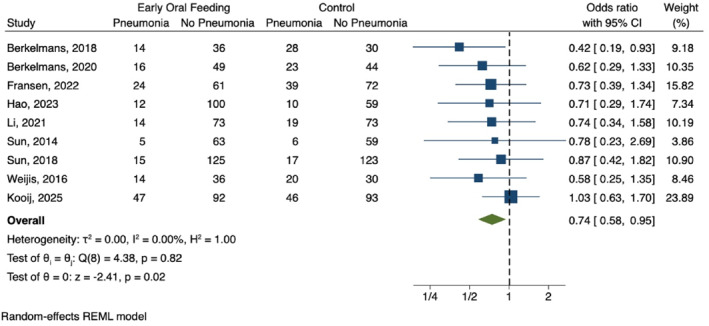
Forrest plot of the random‐effects meta‐analysis for the odds of postoperative pneumonia among studies comparing early oral feeding group to the control group (delayed oral feeding or standard of care) among patients undergoing esophagectomy.

Nine studies reported incidence of postoperative pneumonia (Table 4). Pooled meta‐analysis revealed significantly reduced odds of post‐operative pneumonia among patients in the early oral feeding group compared to standard of care (OR 0.74, 95% CI 0.58–0.95, *p* = 0.02) (Figure 2). Heterogeneity was interpreted as low based on the *I* ^2^ value 0% and the Galbraith plot demonstrating no studies reporting mean estimates outside of the 95% confidence interval of the regression line (Supporting Information S6: Online Resource 6 and Supporting Information S7: Online Resource 7).

### 3.6 Length of Stay

**FIGURE 3:**
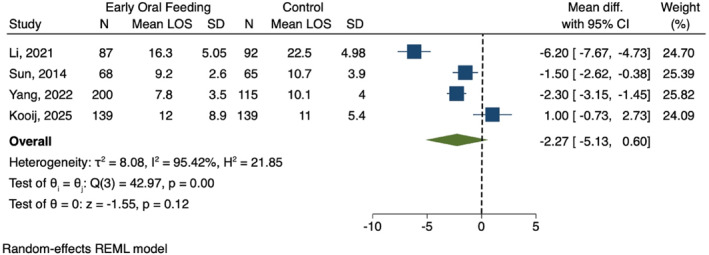
Forrest plot of the random‐effects meta‐analysis for the mean difference of length of stay among studies comparing early oral feeding group to the control group (delayed oral feeding or standard of care) among patients undergoing esophagectomy.

Four studies reported length of stay with mean values and standard deviations (Table 4). Pooled meta‐analysis revealed no significant reduction in length of stay among the early oral feeding group compared to standard of care (MD −2.27, 95% CI –5.13 to 0.60; *p* = 0.12) (Figure 3). Heterogeneity was interpreted as high based on the *I* ^2^ value of 95.4%. The Galbraith plot did not demonstrate any studies reporting mean estimates outside of the 95% confidence interval of the regression line (Supporting Information S8: Online Resource 8 and Supporting Information S9: Online Resource 9).

### 3.7 Publication Bias

Evaluation of publication bias was performed through generating funnel plots, which were evaluated for asymmetry. For anastomotic leak and post‐operative pneumonia, the provided funnel plots (Supporting Information S4: Online Resources 4 and Supporting Information S5: Online Resource 5) do not show evidence of asymmetry, suggesting low publication bias. For length of stay, the provided funnel plot (Supporting Information S6: Online Resource 6), does show evidence of asymmetry suggesting that there is moderate publication bias present, which could be explained by the paucity of studies with appropriate data on length of stay for meta‐analyses.

## 4 Discussion

This systematic review and meta‐analysis evaluated the effect of EOF—defined as oral intake on postoperative day zero or one—after esophagectomy. Across 10 studies involving 1714 patients, EOF was not associated with a statistically significant difference in the odds of anastomotic leak and was linked to reduction in postoperative pneumonia. Though reduction in length of hospital stay (LOS) was not statistically significant, most studies demonstrated a trend toward shorter hospitalization with EOF. All included studies were of moderate‐to‐high quality on the Newcastle–Ottawa Scale. Heterogeneity was low for anastomotic leak and pneumonia outcomes, though high for LOS, likely reflecting variation in discharge protocols and feeding advancement strategies [5, 17, 26].

Anastomotic leak remains one of the most feared complications after esophagectomy, with significant implications for morbidity and mortality. Despite long‐standing concerns that early feeding might impair anastomotic healing, our pooled evidence found no significant difference in leak rates between EOF and standard care. This likely reflects the predominance of liquid diets in the early post‐operative state, which minimizes mechanical stress at the anastomotic site. Most protocols begin with clear or full liquids, with diet advancement based on clinical progress. Of note, patients were carefully screened to exclude those at higher risks (Table 1). A key contemporary trial is NUTRIENT II, which randomized adults undergoing minimally invasive Ivor Lewis esophagectomy with an intrathoracic anastomosis to direct oral feeding versus standard nil‐by‐mouth with tube feeding under standardized definitions and protocols. Complication rates, including leak and pneumonia, were comparable between groups, supporting the feasibility and safety of EOF in carefully selected patients at experienced centers. The trial included consecutive adults (≥ 18 years) scheduled for minimally invasive esophagectomy with intrathoracic Ivor Lewis anastomosis for esophageal cancer, while excluding those with preoperative swallowing disorders or achalasia, malnutrition (> 15% preoperative weight loss), poor functional status (Karnofsky < 80), or inability to tolerate oral intake or receive a feeding jejunostomy. Patients with metastases identified preoperatively or intraoperatively, or those undergoing total gastrectomy, were also excluded, thereby selecting a relatively fit, lower‐risk cohort [22].

The concern that EOF may increase aspiration risk in patients with impaired airway protection was not supported by the meta‐analysis, with a significant reduction in postoperative pneumonia noted (OR 0.74, 95% CI 0.58–0.95, *p* = 0.02). Patient selection likely played a key role: individuals with suspected recurrent laryngeal nerve injury, poor swallowing, or respiratory compromise would have been excluded from EOF protocols (Table 1). Additionally, EOF likely promotes earlier removal of nasogastric tubes and thus earlier mobilization, both of which may improve pulmonary mechanics and reduce infection risk [20]. Nasogastric tubes can impede swallowing reflexes and contribute to reflux or bacterial colonization [27]. Importantly, none of the included studies reported increased rates of aspiration pneumonia, and several observed a trend toward fewer pulmonary complications [6, 22, 28].

Strengths of our study include a rigorous search strategy, inclusion of multiple prospective studies, and a consistent definition of early oral feeding (EOF) as initiation on postoperative day zero or one. This stands in contrast to older systematic reviews, which broadly defined early feeding as initiation up to POD 5 and noted significant heterogeneity across feeding protocols and outcomes [26]. Similarly, a 2025 meta‐analysis by Alrasheed et al. encompassed feeding timelines extending to POD 5 and reported variable definitions that may obscure the true effects of immediate feeding [29]. By incorporating a narrower and clinically relevant definition, our analysis enhances comparability across studies, minimizes heterogeneity, and clarifies the impact of truly early oral feeding interventions. These findings add a stronger and more precise signal to the growing evidence base, supporting EOF as a safe and potentially transformative practice when embedded within structured, multidisciplinary recovery pathways.

Several limitations warrant discussion. Restricting inclusion to studies with EOF on POD zero or one limited the number of papers included for some outcomes, particularly LOS. The nature of oral intake varied—some studies used clear fluids, others allowed full fluids—but most lacked details on diet advancement schedules or the time to solid food. Furthermore, no studies used standardized tools to assess swallowing function across cohorts. The lack of objective assessment likely contributed to variability in patient selection and outcomes. Heterogeneity in surgical approaches, perioperative practices, and baseline patient characteristics (e.g., age, comorbidities, cancer stage) may also have introduced unmeasured confounding, although this was partly mitigated by the inclusion of randomized trials. Furthermore, the results of our meta‐analyses were sustained when we excluded the retrospective studies, as well as when we stratified the analysis by European versus Asian cohorts. Lastly, the small number of LOS studies and potential underreporting of failed EOF attempts or protocol deviations could have influenced estimates.

In conclusion, our findings support the safety and potential benefits of early oral liquid intake in carefully selected patients undergoing esophagectomy. EOF was not associated with increased risk of anastomotic leak or increased LOS and may reduce rates of pneumonia. Patient selection remains essential. Literature suggests that preoperative sarcopenia, smoking, malnutrition, diabetes, and cervical anastomosis are associated with higher complication rates and should inform EOF candidacy [12, 30, 31, 32]. Future prospective studies using standardized feeding protocols and long‐term follow‐up—including readmissions, nutritional recovery, and quality‐of‐life metrics—are warranted. Future prospective studies should focus on refining the selection criteria for patients who may be candidates for early oral feeding so that this modality can safely and routinely be incorporated into post‐operative management strategies. When thoughtfully integrated into ERAS pathways, EOF has the potential to improve recovery, shorten hospitalization, and enhance both surgical outcomes and patient‐centered care in esophageal cancer patients.

## Author Contributions

**Debasri Jena:** investigation, data curation, writing–original draft, visualization. **Lixuan Feng:** investigation, data curation, writing – original draft. **Kwaku Addo‐Osafo:** data curation, writing – original draft, investigation. **Armin Rouhi:** investigation, data curation, writing – review and editing. **Janice Y. Kung:** investigation, data curation, writing – review and editing. **Sukhdeep Jatana:** methodology, validation, formal analysis, writing – review and editing, visualization. **Kevin Verhoeff:** validation, formal analysis, writing – review and editing, supervision. **Uzair Jogiat:** conceptualization, methodology, software, validation, formal analysis, writing – review and editing, visualization, supervision. **Simon R. Turner:** conceptualization, writing – review and editing, supervision, project administration. **Eric L. R. Bédard:** conceptualization, methodology, writing – review and editing, supervision, project administration.

## Funding

The authors have nothing to report.

## Ethics Statement

This research was exempt from research ethics board review at our institution given that it was a systematic review and meta‐analysis.

## Consent

The authors have nothing to report.

## Conflicts of Interest

Simon Turner has a financial relationship with Astra‐Zeneca and Ethicon. Eric L. R. Bédard has a financial relationship with Astra‐Zeneca and Hoffman La Roche. All other authors declared no conflict of interest.

## Supporting information


Supporting Information S1

Supporting Information S2

Supporting Information S3

Supporting Information S4

Supporting Information S5

Supporting Information S6

Supporting Information S7

Supporting Information S8

Supporting Information S9

## Data Availability

The data that supports the findings of this study are available in the supplementary material of this article.
