# Supplementary material for: The Impact of Early Oral Feeding on Post‐Operative Morbidity After Esophagectomy: A Systematic Review and Meta‐Analysis
Source: World J Surg. 2026 May 24;50(7):1982–91. doi: 10.1002/wjs.70417 (PMC13356500; doi:10.1002/wjs.70417)
Supplement: Supplementary file 2 — Supporting Information S2 [file WJS-50-1982-s001.pdf]

The Impact of Early Oral Feeding on Post-operative Morbidity after Esophagectomy: A Systematic Review and Meta-Analysis

Debasri Jena[1], Sabrina Feng BSc[1], Kwaku Addo-Osafu MSc[1], Armin Rouhi MD[1,2], Janice Y Kung[3], Sukhdeep Jatana MD[1,2], Kevin Verhoeff MD PhD[1,2], Uzair Jogiat MD PhD[1,2], Simon R. Turner MD MEd[1,2], Eric LR Bédard MD MSc[1,2]*

[1]Division of Thoracic Surgery, University of Alberta, Edmonton, Alberta, Canada.

[2]Department of General Surgery, University of Alberta, Edmonton, Alberta, Canada.

[3]Geoffrey and Robyn Sperber Health Sciences Library, University of Alberta, Edmonton, Alberta, Canada

***Corresponding Author**:

Eric LR Bédard

Division of Thoracic Surgery, Room 4-417, Community Services Center, Royal Alexandra Hospital, 10240 Kingsway Avenue, Edmonton, Alberta, T5H3V9, Canada

E-mail: ebedard@ualberta.ca

Online Resource 2. Search Strategy

Database	Search Strategy
**MEDLINE**<br><br>Ovid MEDLINE(R) ALL 1946 to October 01, 2025	1. exp Esophagectomy/ or (oesophagectom* or esophagectom*).mp.<br>2. Ivor Lewis.tw,kf.<br>3. colonic interposition.mp.<br>4. ((esophag* or oesophag*) adj3 resection*).mp.<br>5. *Esophageal Neoplasms/su [Surgery]<br>6. exp Esophageal Neoplasms/rh [Rehabilitation]<br>7. exp Esophageal Achalasia/su [Surgery]<br>8. or/1-7<br>9. exp Enteral Nutrition/mt [Methods]<br>10. ((oral* or post-operative* or postoperative*) adj3 (feed* or nutrition or diet)).mp.<br>11. (clear fluid* or full fluid* or regular diet*).mp.<br>12. "diet as tolerated".mp.<br>13. or/9-12<br>14. 8 and 13<br>15. limit 14 to english language
**Embase**<br><br>Ovid Embase 1974 to 2025 September 30	1. exp esophagectomy/ or (oesophagectom* or esophagectom*).mp.<br>2. Ivor Lewis.tw,kw.<br>3. colonic interposition.mp.<br>4. ((esophag* or oesophag*) adj3 resection*).mp.<br>5. *esophagus tumor/su [Surgery]<br>6. exp esophagus tumor/rh [Rehabilitation]<br>7. exp esophagus achalasia/su [Surgery]<br>8. or/1-7<br>9. ((oral* or post-operative* or postoperative*) adj3 (feed* or nutrition or diet)).mp.<br>10. (clear fluid* or full fluid* or regular diet*).mp.<br>11. "diet as tolerated".mp.<br>12. or/9-11<br>13. 8 and 12

	14. limit 13 to english language
**Cochrane Library**	#1 [mh Esophagectomy] or oesophagectom* or esophagectom*<br>#2 Ivor Lewis:ti,ab,kw<br>#3 colonic interposition<br>#4 ((esophag* or oesophag*) NEAR/3 resection*)<br>#5 MeSH descriptor: [Esophageal Neoplasms] explode all trees<br>#6 MeSH descriptor: [Esophageal Achalasia] explode all trees and with qualifier(s): [surgery - SU]<br>#7 {OR #1-#6}<br>#8 MeSH descriptor: [Enteral Nutrition] explode all trees and with qualifier(s): [methods - MT]<br>#9 ((oral* or post-operative* or postoperative*) NEAR/3 (feed* or nutrition or diet))<br>#10 (clear NEXT fluid*) or (full NEXT fluid*) or (regular NEXT diet*)<br>#11 diet as tolerated<br>#12 {OR #8-#11}<br>#13 #7 AND #12<br><br>Limit: English language
**Scopus**	(TITLE-ABS-KEY (oesophagectom* OR esophagectom* OR "Ivor Lewis " OR "colonic interposition " OR ((esophag* OR oesophag*) W/3 resection*)) AND TITLE-ABS-KEY (((oral* OR post-operative* OR postoperative*) W/3 (feed* OR nutrition OR diet)) OR "clear fluid*" OR "full fluid*" OR "regular diet*" OR "diet as tolerated")) AND (LIMIT-TO (LANGUAGE , "English"))
**Web of Science Core Collection**	TS=(oesophagectom* OR esophagectom* OR "Ivor Lewis " OR "colonic interposition " OR ((esophag* OR oesophag*) NEAR/3 resection*)) AND TS= (((oral* OR post-operative* OR postoperative*) NEAR/3 (feed* OR nutrition OR diet)) OR "clear fluid*" OR "full fluid*" OR "regular diet*" OR "diet as tolerated")<br><br>Refined by: English language
**Google Scholar**	(oesophagectomy OR esophagectomy OR "Ivor Lewis" or "colonic interposition" or "esophageal resection") AND (oral feeding OR post-operative nutrition OR postoperative diet OR "clear fluid" OR "full fluid" OR

	"regular diet" OR "diet as tolerated")
