# Supplementary material for: The Impact of Early Oral Feeding on Post‐Operative Morbidity After Esophagectomy: A Systematic Review and Meta‐Analysis
Source: World J Surg. 2026 May 24;50(7):1982–91. doi: 10.1002/wjs.70417 (PMC13356500; doi:10.1002/wjs.70417)

The Impact of Early Oral Feeding on Post-operative Morbidity after Esophagectomy: A Systematic Review and Meta-Analysis

Debasri Jena[1], Sabrina Feng BSc[1], Kwaku Addo-Osafu MSc[1], Armin Rouhi MD[1,2], Janice Y Kung[3], Sukhdeep Jatana MD[1,2], Kevin Verhoeff MD PhD[1,2], Uzair Jogiat MD PhD[1,2], Simon R. Turner MD MEd[1,2], Eric LR Bédard MD MSc[1,2]*

[1]Division of Thoracic Surgery, University of Alberta, Edmonton, Alberta, Canada.

[2]Department of General Surgery, University of Alberta, Edmonton, Alberta, Canada.

[3]Geoffrey and Robyn Sperber Health Sciences Library, University of Alberta, Edmonton, Alberta, Canada

***Corresponding Author**:

Eric LR Bédard

Division of Thoracic Surgery, Room 4-417, Community Services Center, Royal Alexandra Hospital, 10240 Kingsway Avenue, Edmonton, Alberta, T5H3V9, Canada

E-mail: ebedard@ualberta.ca

Online Resource 8. Galbraith plot for evaluation of heterogeneity in the random-effects meta-analysis for the mean difference in length of stay.

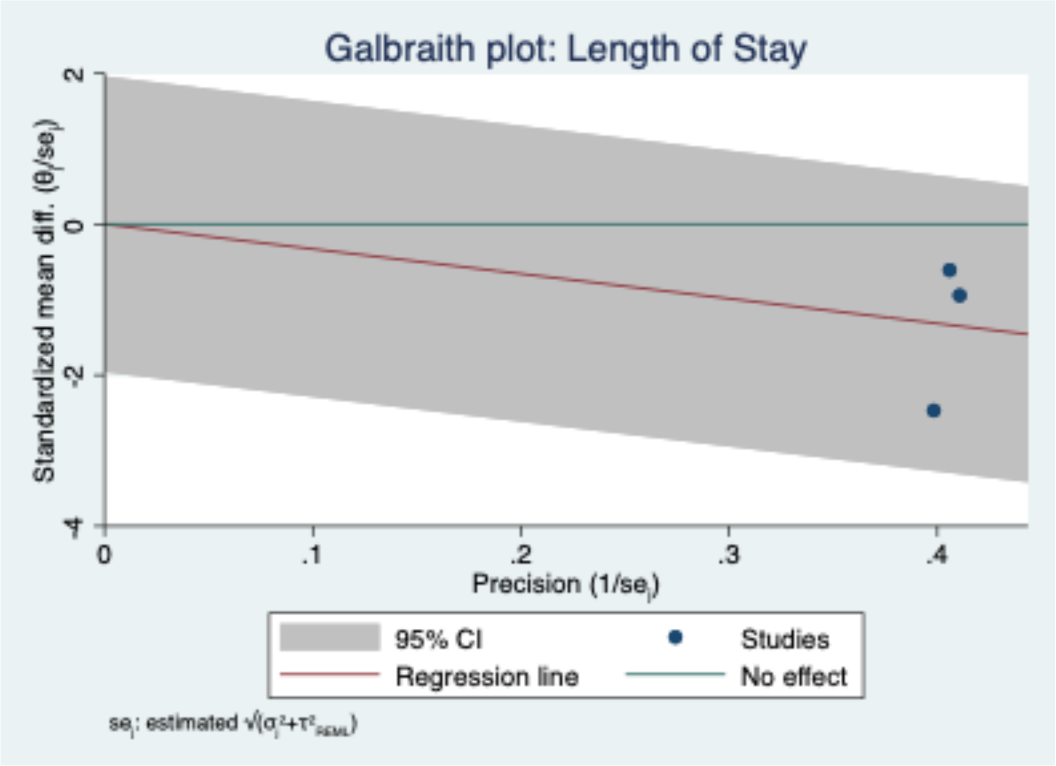

Supplement: Supplementary file 8 — Supporting Information S8 [file WJS-50-1982-s006.pdf]
